# Interplay among *Drosophila* transcription factors Ets21c, Fos and Ftz-F1 drives JNK-mediated tumor malignancy

**DOI:** 10.1242/dmm.020719

**Published:** 2015-10-01

**Authors:** Eva Külshammer, Juliane Mundorf, Merve Kilinc, Peter Frommolt, Prerana Wagle, Mirka Uhlirova

**Affiliations:** 1 Institute for Genetics and Cologne Excellence Cluster on Cellular Stress Responses in Aging-Associated Diseases (CECAD), University of Cologne, 50931 Cologne, Germany; 2 Bioinformatics Facility, CECAD Research Center, University of Cologne, 50931 Cologne, Germany

**Keywords:** Oncogene cooperation, Transcription factors, *Drosophila*, Epithelia, Cancer, Jun-N-terminal kinase, JNK

## Abstract

Cancer initiation and maintenance of the transformed cell state depend on altered cellular signaling and aberrant activities of transcription factors (TFs) that drive pathological gene expression in response to cooperating genetic lesions. Deciphering the roles of interacting TFs is therefore central to understanding carcinogenesis and for designing cancer therapies. Here, we use an unbiased genomic approach to define a TF network that triggers an abnormal gene expression program promoting malignancy of clonal tumors, generated in *Drosophila* imaginal disc epithelium by gain of oncogenic Ras (Ras^V12^) and loss of the tumor suppressor Scribble (*scrib^1^*). We show that malignant transformation of the *ras^V12^scrib^1^* tumors requires TFs of distinct families, namely the bZIP protein Fos, the ETS-domain factor Ets21c and the nuclear receptor Ftz-F1, all acting downstream of Jun-N-terminal kinase (JNK). Depleting any of the three TFs improves viability of tumor-bearing larvae, and this positive effect can be enhanced further by their combined removal. Although both Fos and Ftz-F1 synergistically contribute to *ras^V12^scrib^1^* tumor invasiveness, only Fos is required for JNK-induced differentiation defects and Matrix metalloprotease (MMP1) upregulation. In contrast, the Fos-dimerizing partner Jun is dispensable for JNK to exert its effects in *ras^V12^scrib^1^* tumors. Interestingly, Ets21c and Ftz-F1 are transcriptionally induced in these tumors in a JNK- and Fos-dependent manner, thereby demonstrating a hierarchy within the tripartite TF network, with Fos acting as the most upstream JNK effector. Of the three TFs, only Ets21c can efficiently substitute for loss of polarity and cooperate with Ras^V12^ in inducing malignant clones that, like *ras^V12^scrib^1^* tumors, invade other tissues and overexpress MMP1 and the *Drosophila* insulin-like peptide 8 (Dilp8). While *ras^V12^ets21c* tumors require JNK for invasiveness, the JNK activity is dispensable for their growth. In conclusion, our study delineates both unique and overlapping functions of distinct TFs that cooperatively promote aberrant expression of target genes, leading to malignant tumor phenotypes.

## Abstract (precis)

**Summary:** This study provides genetic evidence that malignancy driven by oncogenic Ras and loss of polarity requires transcription factors of three distinct protein families, acting in synergy downstream of JNK signaling.

## INTRODUCTION

The emergence of tumors in a formerly healthy organ is a multistep process, during which transformed cells unleash their growth and proliferative potential, circumvent apoptosis, invade adjacent tissues and disseminate. The acquisition of such hallmarks of cancer results from malfunction of cellular signaling circuits and aberrant gene expression induced via cooperating oncogenic lesions (Hanahan and Weinberg, 2011). A majority of signaling pathways converge on transcription factors (TFs) that control cell function and homeostasis through binding to specific DNA sequences and orchestrating gene expression programs. Indeed, TFs are often functionally altered in diverse human malignancies, frequently acting as oncoproteins or tumor suppressors (Darnell, 2002). Among TFs recurrently implicated in human cancers are members of three protein families: nuclear receptors (NRs); ETS-domain proteins; and the basic leucine zipper (bZIP) factors (Sharrocks, 2001; Eferl and Wagner, 2003; Ahmad and Kumar, 2011). The latter form homo- or heterodimeric transcription-activating complexes, such as the prototypical activating protein 1 (AP-1) consisting of proteins of the Jun and Fos families (Kockel et al., 2001; Hess et al., 2004).

Analyses of candidate genes and genome-wide approaches using cancer cell lines or tumor samples have shown that TFs act through combinatorial mutual interactions on overlapping sets of target genes. For example, AP-1 motifs adjacent to ETS binding sites are overrepresented within regulatory sequences of genes required for migration of cells transformed by the activated Ras proto-oncogene (Plotnik et al., 2014) or in the promoter of the uridine phosphorylase (UPP) gene, whose ectopic expression supports anchorage-independent growth of cells overexpressing the EWS-ETS fusion oncoprotein (Deneen et al., 2003; Kim et al., 2006). How individual TFs and their interplay contribute to tumor development and malignancy *in vivo* is far less clear owing to the high degree of genetic redundancy and the technical and ethical obstacles associated with generating and manipulating conventional mammalian cancer models.

The fruit fly *Drosophila melanogaster* has been used extensively to decipher the roles of TFs of distinct families in development and physiology. Genetic studies in *Drosophila* led to the initial discovery of the Pointed (PNT) domain in the ETS transcription factor Pointed (Klämbt, 1993) and inspired research on regulation and function of *ets* genes (Sharrocks, 2001). Analyses of *Drosophila* embryos that remain dorsally open as a result of mutations in either the *jun* (*jun related antigen*, *jra*) or *fos* (*kayak*, *kay*) genes, have established the Jun/Fos heterodimer as a key regulator of epithelial cell morphogenesis (Kockel et al., 2001). Genetic analyses of *Drosophila* NRs have identified their role in controlling major developmental transition and maturation (King-Jones and Thummel, 2005). Furthermore, recent advances in genomics and proteomics allow large-scale mapping of DNA binding sites for TFs (Adryan and Teichmann, 2006; Hens et al., 2011; Shazman et al., 2013; Nitta et al., 2015) and TF protein interaction networks (Rhee et al., 2014).

Importantly, during the last decade, *Drosophila* has become instrumental to our understanding of the mechanisms of cancer initiation and progression, revealing novel molecular components and signaling networks (Miles et al., 2011; Stefanatos and Vidal, 2011; Gonzalez, 2013). Tumor development can be recapitulated in flies by combining defined somatic gain- and loss-of-function mutations in clones of marked cells within the eye/antennal imaginal disc (EAD) epithelium. While overexpression of the oncogenic form of Ras (Ras^V12^) alone results in hyperplasia and ectopic differentiation, combination of *ras^V12^* with loss of polarity regulators, such as the neoplastic tumor suppressor gene *scribble* (*scrib*), transforms the clones into highly malignant, deadly tumors. These proliferate without differentiating, resist apoptosis, lose polarity, induce inflammation and invade neighboring tissues (Brumby and Richardson, 2003; Pagliarini and Xu, 2003; Pastor-Pareja et al., 2008; Cordero et al., 2010). The invasion of *ras^V12^scrib^1^* tumors depends strictly on aberrant activation of Jun-N-terminal kinase (JNK) signaling by loss of epithelial polarity (Brumby and Richardson, 2003; Igaki et al., 2006; Uhlirova and Bohmann, 2006).

**TRANSLATIONAL IMPACT**

**Clinical issue**

Transcriptional regulation of gene expression is fundamental to organismal development and homeostasis. In response to extracellular signals, appropriate gene expression programs are activated by combinatorial interactions among transcription factors (TFs). Incorrect TF activities accompany progressive stages of malignant transformation. Although TFs were originally thought to be undruggable, a recently revived effort to design anti-cancer drugs that target specific TFs is showing promise. Unraveling the roles of individual TFs and their interactions is therefore central to combating cancer.

**Results**

In this study, the authors applied a genomic approach to characterize gene expression changes and TF networks underlying malignancy of tumors that are induced in the developing *Drosophila* epithelium by defined oncogenic lesions. They provide genetic evidence that malignant transformation in this model requires three TFs, namely Fos, Ets21c and Ftz-F1, homologs of which have been implicated in different types of human cancer. They demonstrate both unique and synergistic roles for these TFs in promoting differentiation defects and invasiveness of the tumors *in vivo*.

**Implications and future directions**

Given the conserved nature of these proteins, it is likely that this tripartite network of TFs also operates in human disease. Further characterization of complex TF interactions in the simple and genetically tractable *Drosophila* model opens a unique avenue to deciphering the contribution of TF cooperation and aberrant gene expression programs during malignant transformation. The design of therapeutics targeting these essential cooperating TFs at the nexus of pathways fundamental to cancer progression might improve the chances of recovery for patients.

While significant attention has been devoted to mechanisms that activate JNK upon polarity disruption (Igaki et al., 2006; Cordero et al., 2010; Brumby et al., 2011; Jiang et al., 2011), less is known about TFs that translate JNK activity into changes in gene expression. We have shown previously that Fos is required downstream of JNK to promote cell migration and tumor cell invasiveness by upregulating Matrix metalloprotease 1 (MMP1) and the actin cross-linking protein FilaminA/Cheerio, which cooperatively disorganize epithelia, allowing cells to breach the basement membrane and spread to secondary sites (Uhlirova and Bohmann, 2006; Külshammer and Uhlirova, 2013). As expression patterns of most genes in eukaryotes are determined by an interplay among several TFs (Halfon et al., 2002), the complex response elicited by JNK in the context of malignant *ras^V12^scrib^1^* tumors must involve several transcription regulators in addition to Fos.

Here, we show that in the invasive *ras^V12^scrib^1^* tumors, JNK signaling induces dramatic changes to the gene expression program through specific TFs that belong to diverse families. The nuclear receptor Ftz-F1, the ETS-domain transcription factor Ets21c and the bZIP protein Fos all exert unique and overlapping functions in promoting full malignancy of the *ras^V12^scrib^1^* tumors, but only Ets21c is sufficient to induce malignant tumors in cooperation with activated Ras. Our study thus delineates a transcription factor network that alters target gene expression and promotes tumor phenotypes in response to aberrant Ras and JNK signaling.

## RESULTS

### Malignant *ras^V12^scrib^1^* tumors exhibit a unique gene-expression profile

To obtain a complete picture of gene expression changes during distinct stages of tumorigenesis, we deep sequenced RNA libraries prepared from *Drosophila* third-instar larval EAD bearing clones of normal (control) and tumor cells of defined genotypes. The tumors were benign *ras^V12^*, malignant *ras^V12^scrib^1^* or malignant yet non-invasive *ras^V12^scrib^1^bsk^DN^*, where JNK was inactivated by expression of its dominant-negative form, Bsk^DN^ (supplementary material Table S1). This approach allowed us to identify genes that were differentially regulated (≥1.5-fold change) in the tumors of distinct malignancy relative to control.

**Fig. 1.**
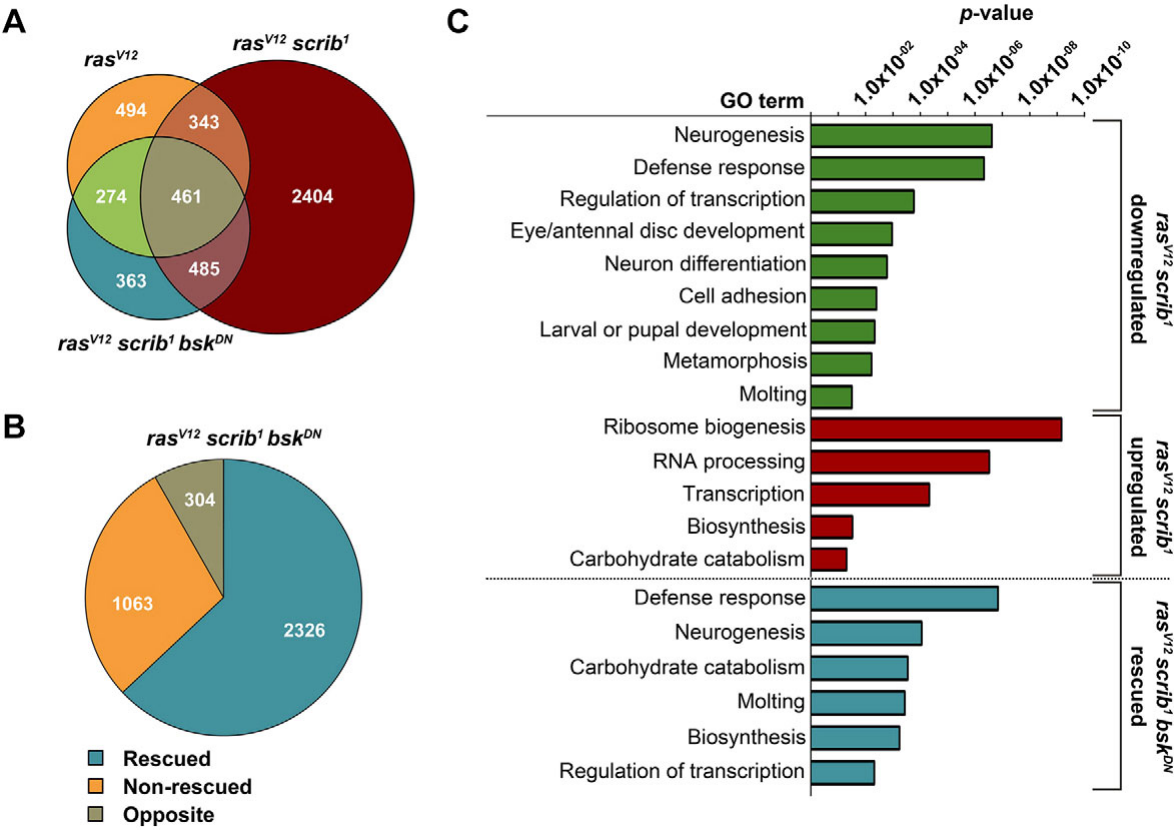
**Malignant *ras^V12^scrib^1^* tumors exhibit a unique gene expression profile.** (A) Venn diagram shows marked increase in number of genes whose expression changed ≥1.5-fold relative to control (*P*<0.05) in the EAD bearing *ras^V12^scrib^1^* (in total 3693 genes) compared with *ras^V12^* alone (1572 genes). Inhibition of JNK signaling (*ras^V12^scrib^1^bsk^DN^*) reduced the number of deregulated transcripts to 1583. (B) Blocking JNK activity rescued 63% of deregulated genes (blue) in *ras^V12^scrib^1^* tumors, with rescue defined as ≥1.5-fold change in expression from *ras^V12^scrib^1^* towards control levels. Non-invasive *ras^V12^scrib^1^bsk^DN^* tumors also exhibited a unique set of genes (8%) regulated in a direction opposite to *ras^V12^scrib^1^* mosaic EAD. (C) Distinct functional GO clusters enriched among genes ectopically expressed (red) or downregulated (green) in *ras^V12^scrib^1^* tumors and among those rescued in *ras^V12^scrib^1^bsk^DN^* (blue) identified by DAVID*.* For genes falling into individual GO categories, see supplementary material Table S1.

While constitutive activation of Ras signaling (*ras^V12^*) alone altered expression of 1572 transcripts, additional loss of the apico-basal polarity gene *scribble* (*ras^V12^scrib^1^*) dramatically increased the number to 3693 (Fig. 1A). Inhibition of JNK signaling (*ras^V12^scrib^1^bsk^DN^*) reduced the number of deregulated genes to 1583 (Fig. 1A). Comparison of the tumor transcriptomes revealed 2404 distinct mRNAs that were specifically altered only in the EAD bearing invasive *ras^V12^scrib^1^* tumors (Fig. 1A). Strikingly, expression of 63% of all mRNAs deregulated in *ras^V12^scrib^1^* tumors was ‘rescued’ towards control levels when JNK was inhibited (*ras^V12^scrib^1^bsk^DN^*; Fig. 1B). These data demonstrate the vast impact of aberrant JNK activity on tumor transcriptome and indicate that changes to gene expression elicited by JNK are the mechanism underlying JNK-mediated malignancy. However, in addition to normalizing expression of many tumor-signature transcripts, the *ras^V12^scrib^1^bsk^DN^* clones also exhibited a unique profile, with 304 genes that were regulated in the opposite direction from those in the *ras^V12^scrib^1^* clones (Fig. 1B) and 363 genes that were misexpressed exclusively in *ras^V12^scrib^1^bsk^DN^* tumors (Fig. 1A).

The follow-up gene ontology (GO) clustering analysis using DAVID (Huang et al., 2009) revealed that genes associated with ‘neurogenesis’, ‘neuron differentiation’ and ‘metamorphosis’ were markedly enriched among transcripts downregulated in *ras^V12^scrib^1^* tumors (Fig. 1C, supplementary material Table S1). These data conform well to phenotypes of larvae bearing *ras^V12^scrib^1^* clonal tumors, including their inability to pupate and undergo metamorphosis as well as failure of neoplastic *ras^V12^scrib^1^* cells to differentiate into photoreceptors (Brumby and Richardson, 2003; Uhlirova and Bohmann, 2006). In contrast, genes upregulated in *ras^V12^scrib^1^* tumors were associated with the GO terms ‘ribosome biogenesis’, ‘RNA processing’, ‘biosynthesis’ and ‘carbohydrate catabolism’ (Fig. 1C), reflecting increased demand for macromolecule biosynthesis to support tumor cell growth and division. Enrichment of the GO cluster related to ‘transcription’ matched the highly aberrant gene expression program of tumor cells. Strikingly, similar clusters, including ‘neurogenesis’, ‘regulation of transcription’, ‘molting’ and ‘biosynthesis’, were identified as rescued in *ras^V12^scrib^1^bsk^DN^* compared with *ras^V12^scrib^1^* clones (Fig. 1C). These data are consistent with previously demonstrated recovery of photoreceptor differentiation, suppressed invasiveness and restored pupation in larvae bearing *ras^V12^scrib^1^bsk^DN^* tumors (Uhlirova and Bohmann, 2006; Srivastava et al., 2007; Leong et al., 2009; Külshammer and Uhlirova, 2013).

### A transcription factor network underlies tumor-specific gene-expression signature

To decipher which TFs might be responsible for the tumor-specific expression signatures, we searched for putative TF binding sites enriched among genes differentially regulated in the different tumor types using the iRegulon Cytoscape plugin (Shannon et al., 2003; Janky et al., 2014).

**Fig. 2.**
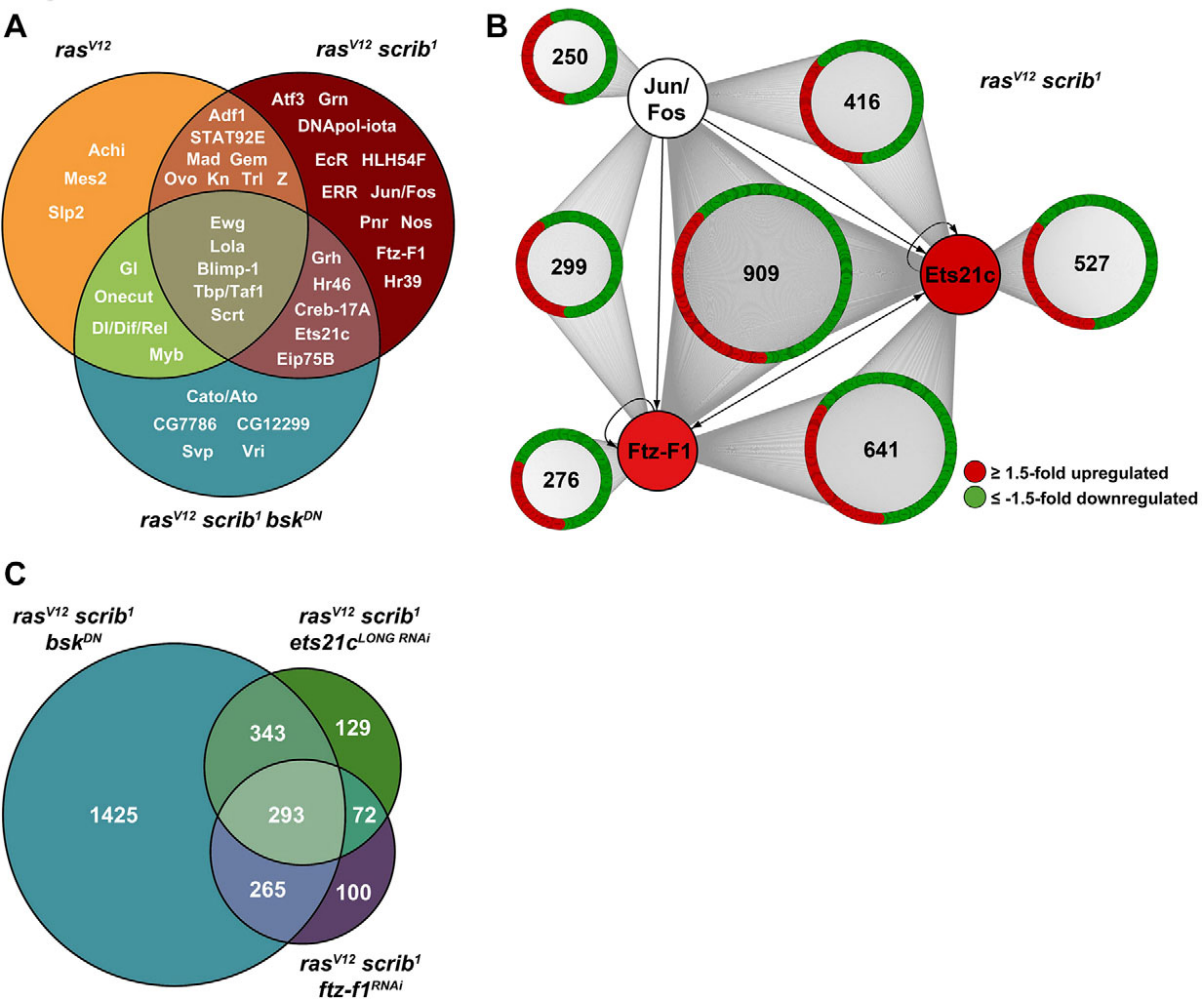
**Transcription factor network orchestrates tumor-specific gene expression signature.** (A) The number and diversity of enriched DNA motifs and hence putative TFs that regulate gene expression increase with tumor complexity as identified by iRegulon. Venn diagram shows specific enrichment of the binding sites for AP-1 factors (e.g. Jun/Fos), Atf3, NRs (e.g. Ftz-F1, EcR) in *ras^V12^scrib^1^* mosaic EAD, whereas an Ets21c motif is overrepresented also among genes regulated in *ras^V12^scrib^1^bsk^DN^* mosaic EAD. (B) Putative AP-1, Ftz-F1 and Ets21c binding motifs were found by FIMO 5 kb upstream and within first introns of numerous genes misregulated in *ras^V12^scrib^1^* tumors. Of those genes, many contain binding sites for all three TFs or a combination of Ets21c/Ftz-F1 or Ets21c/AP-1 motifs. The network connects the candidate TFs to their putative target genes that are up- (red) or downregulated (green; ≥1.5-fold) in *ras^V12^scrib^1^* tumors. In contrast to Jun and Fos, Ftz-F1 and Ets21c are themselves transcriptionally upregulated in *ras^V12^scrib^1^* malignant tumors, possibly through a self-regulatory and/or AP-1-dependent mechanism (arrows). (C) Venn diagram shows intersection of genes that are misregulated in *ras^V12^scrib^1^* tumors but rescued upon inhibition of JNK (*ras^V12^scrib^1^bsk^DN^*), knockdown of *ets21c* (*ras^V12^scrib^1^ets21c^LONG RNAi^*) or *ftz-f1* (*ras^V12^scrib^1^ftz-f1^RNAi^*). Rescue was defined as ≥1.5-fold change in expression from *ras^V12^scrib^1^* towards control levels. (B,C) See supplementary material Table S1 for corresponding gene lists.

In contrast to few motifs (e.g. Achi, Mes2, Slp2) exclusive to the transcriptome of non-invasive *ras^V12^* tumors (Fig. 2A), we identified numerous distinct DNA elements among genes regulated in *ras^V12^scrib^1^* tumors bound by TFs of different families, including STAT (Stat92E), GATA (Grn, Pnr), bHLH (HLH54F), ETS (Ets21c), BTB (Lola), bZIP proteins (Atf3, Fos, Jun, Creb-17A) and NRs (Hr39, Eip75B, EcR, Hr46, Ftz-F1; Fig. 2A). Such a dramatic increase in the number and diversity of binding motifs strongly suggested that the expression profile of *ras^V12^scrib^1^* tumors resulted from a cooperative network of multiple TFs as opposed to the activity of one particular TF. Upon JNK inhibition, the diversity of binding motifs was greatly reduced (Fig. 2A) as the number of deregulated genes decreased relative to *ras^V12^scrib^1^* clones (Fig. 1A), thus implicating JNK as a master regulator of those TFs that cooperatively drive the altered *ras^V12^scrib^1^* tumor transcriptome. Indeed, the AP-1 elements recognized by dimers of bZIP TFs, such as Jun and Fos, in response to JNK activation were enriched exclusively in the *ras^V12^scrib^1^* data set (Fig. 2A).

The requirement for Fos in the JNK-mediated invasiveness of *ras^V12^scrib^1^* tumors has been demonstrated previously (Uhlirova and Bohmann, 2006; Külshammer and Uhlirova, 2013). However, except for a recently reported involvement of Stat92E in control of *ras^V12^scrib^1^* tumor growth (Davie et al., 2015), the roles of Jun and other TFs, predicted from our data sets, are unknown. Therefore, we next focused on Jun and two other proteins whose orthologs have been associated with human cancer: the ETS-domain transcription factor Ets21c and the nuclear receptor Fushi tarazu transcription factor 1 (Ftz-F1; Fig. 2A). *Drosophila ets21c* has been described as an immune-regulated gene induced in response to immune challenge and wounding (Boutros et al., 2002; Park et al., 2004; Radyuk et al., 2010; Chambers et al., 2012; Patterson et al., 2013), whereas Ftz-F1 is a founding member of the NR5A family of nuclear receptors with an essential function in segmentation and metamorphosis (Pick et al., 2006).

### *ets21c* and *ftz-f1* transcripts are regulated in a JNK-Fos-dependent manner

In contrast to *fos* and *jun* mRNAs, whose levels remained unchanged, expression of *ets21c* and *ftz-f1* was elevated in *ras^V12^scrib^1^* tumors (supplementary material Table S1, Fig. S1). The *ets21c* and *ftz-f1* genomic loci each encode two protein isoforms with different N-termini encoded by alternative first exons (The FlyBase Consortium, 2003). Quantitative real-time PCR (qRT-PCR) revealed that the marked increase of *ets21c* mRNA in *ras^V12^scrib^1^* tumors could be ascribed mainly to the *ets21c-RA* isoform (hereafter *ets21c^LONG^*), whereas upregulation of *ets21c-RB* (*ets21c^SHORT^*) was minor (supplementary material Fig. S1). In contrast, *α-ftz-f1* and *β-ftz-f1* isoforms were both upregulated in *ras^V12^scrib^1^* tumors to a similar extent (supplementary material Fig. S1). All four transcripts returned close to control levels upon inhibition of JNK or loss of TF Fos (supplementary material Fig. S1).

To assess the potential impact of individual TFs on the *ras^V12^scrib^1^* tumor transcriptome, we employed the FIMO (Find Individual Motif Occurrence) online tool (Bailey et al., 2009; Grant et al., 2011). We scanned the selected regions of all 3693 differentially expressed genes for the presence of the AP-1, Ets21c and Ftz-F1 DNA binding motifs. While the Ets21c motif was highly abundant, occurrence of sites for Ftz-F1 or AP-1 appeared restricted. However, none of the motifs associated preferentially with genes regulated in a particular direction (Fig. 2B). Interestingly, a large fraction of the genes contained binding sites for all three TFs or a combination of Ets21c/Ftz-F1 or Ets21c/AP-1 sites (Fig. 2B, supplementary material Table S1). Taken together, these data show that Ets21c and Ftz-F1 are transcriptionally induced in a JNK-Fos-dependent manner and predict that cooperation and/or competition among AP-1, Ets21c and Ftz-F1 contributes to the transcriptome changes and tumor phenotypes in *ras^V12^scrib^1^* clones.

### Suppression of *ftz-f1* and *ets21c* partly recapitulates the transcriptome profile of JNK-depleted, non-invasive tumors

Having established *ets21c* and *ftz-f1* as targets of JNK-Fos signaling, we hypothesized that inhibiting the function of either gene in *ras^V12^scrib^1^* clones using RNAi should recapitulate, at least in part, the transcriptional signature of the *ras^V12^scrib^1^bsk^DN^* mosaic EAD. Furthermore, unbiased profiling of transcriptomes from these EAD should determine whether genes identified by an *in silico* approach are indeed regulated by the specific TFs in the tumor context. Based on the presence of *ets21c* and *ftz-f1* mRNAs in *ras^V12^scrib^1^* clones (supplementary material Fig. S1), we used RNAi lines targeting the *ets21c^LONG^* isoform (*ets21c^LONG RNAi^*) or both *α-ftz-f1* and *β-ftz-f1* transcripts (*ftz-f1^RNAi^*). Knockdown of either *ets21c^LONG^* or *ftz-f1* alone in EAD clones did not affect normal eye/antennal development (supplementary material Fig. S2A-C). RNA sequencing revealed that 22% of predicted Ets21c targets and 17% of putative Ftz-F1 targets, respectively, were altered in their expression in *ras^V12^scrib^1^ets21c^LONG RNAi^* and *ras^V12^scrib^1^ftz-f1^RNAi^* relative to *ras^V12^scrib^1^* tumors. Importantly, genes whose expression was normalized by inhibiting JNK in the *ras^V12^scrib^1^* background (Fig. 1B, supplementary material Table S1) overlapped with transcripts rescued by the removal of Ets21c or Ftz-F1 from the tumors, such that 293 mRNAs were commonly regulated in *ras^V12^scrib^1^bsk^DN^*, *ras^V12^scrib^1^ets21c^LONG RNAi^* and *ras^V12^scrib^1^ftz-f1^RNAi^* transcriptomes (Fig. 2C). Although this overlap in rescued genes provides further support for the action of Ets21c and Ftz-F1 downstream of JNK signaling, the gene-expression signatures of EAD tumors lacking JNK, Ets21c and Ftz-F1 functions are not identical, implying unique JNK-independent roles for Ets21c and Ftz-F1.

### Both Ets21c and Ftz-F1 are required for tumorigenesis

**Fig. 3.**
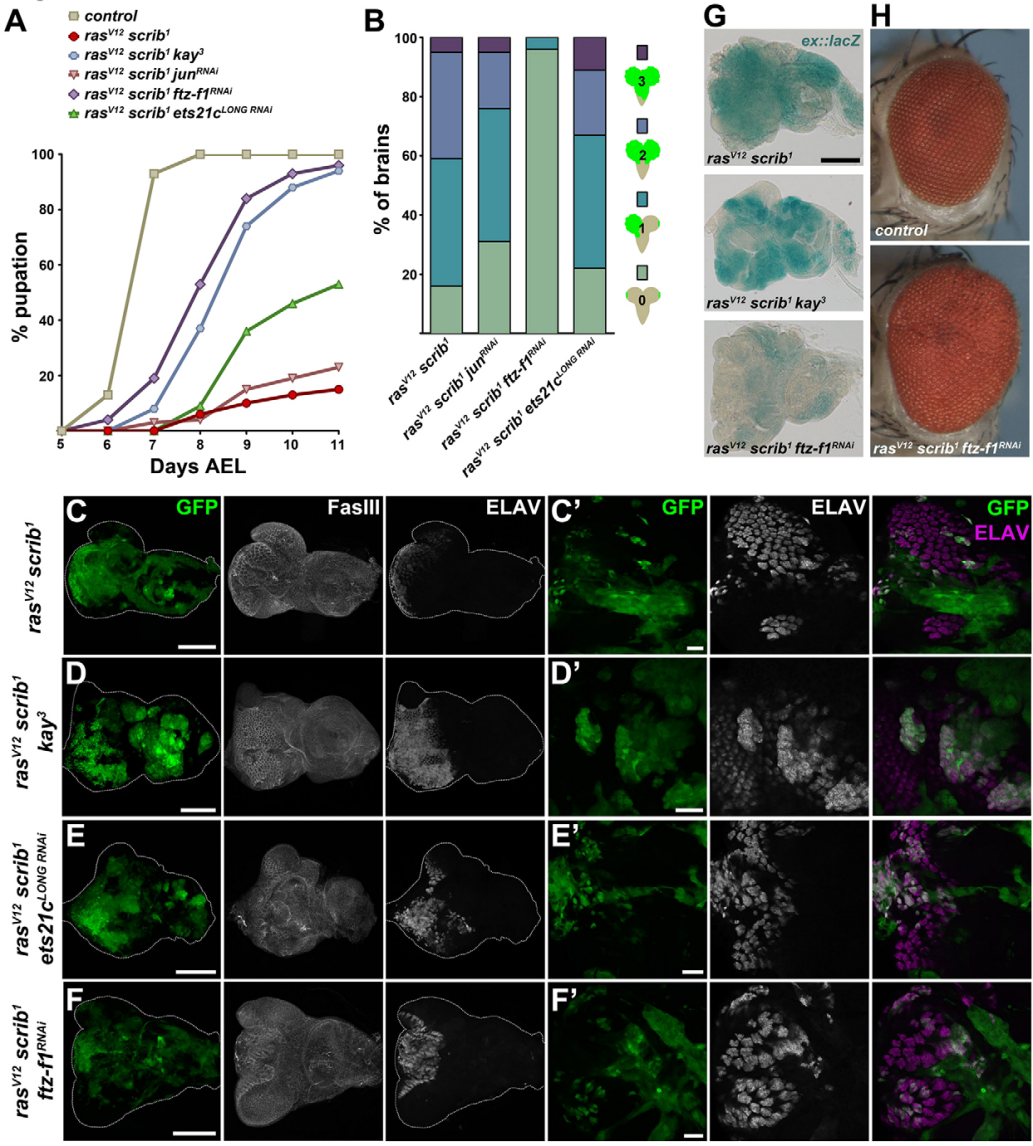
**Both Ftz-F1 and Ets21c are required for tumorigenesis.** (A) Whereas control larvae underwent pupariation on day 6-7 AEL, most of the animals bearing *ras^V12^scrib^1^* EAD tumors died as giant larvae, only rarely forming pseudopuparia. Interfering with Fos*,* Ftz-F1 or Ets21c^LONG^ function in *ras^V12^scrib^1^* clones markedly improved pupation rate, whereas *jun* depletion had no effect. The timing of the larval-pupal transition was partly rescued upon *ftz-f1* RNAi and loss of *fos* (*kay^3^*)*.* The graph shows the cumulative percentage of pupae forming over time. All genotypes differed significantly from control, and except *jun^RNAi^*, also from *ras^V12^scrib^1^* (*P*<0.0001). (B) Reducing *ftz-f1*, but not *jun* or *ets21c^LONG^*, significantly suppressed tumor invasiveness (*P*<0.001). Four grades of invasiveness were scored based on spreading of clonal GFP-positive cells into larval brains dissected on day 7 AEL. Results are the percentage of brains falling into each category. (C-F) Loss of *fos* or knockdown of *ets21c^LONG^* in *ras^V12^scrib^1^* tumors did not affect size of the GFP-labeled clones, whereas *ftz-f1^RNAi^* slightly reduced the tumor burden. The EAD morphology was visualized by immunostaining against Fasciclin III (C-F). Overgrowing *ras^V12^scrib^1^* cells failed to differentiate into photoreceptors, as shown by loss of ELAV staining (C′). Few ELAV-positive cells were detected in *ras^V12^scrib^1^ftz-f1^RNAi^* and *ras^V12^scrib^1^ets21c^LONG RNAi^* discs, which formed greatly disorganized ommatidial clusters (E′,F′), whereas many more *ras^V12^scrib^1^kay^3^* cells differentiated (D′). All images show EAD dissected 6 days AEL, either as projections of multiple confocal sections (C-F) or as single sections (C′-F′). Scale bars: 100 µm (C-F) and 20 µm (C′-F′). (G) Activity of the *ex::lacZ* reporter is markedly lowered upon inhibition of *ftz-f1* but not *fos* in *ras^V12^scrib^1^* clones of EAD. All samples were stained for the same period of time. (H) Thirteen per cent of the *ras^V12^scrib^1^ftz-f1^RNAi^* tumor-bearing animals eclosed as adults with enlarged, rough eyes.

To demonstrate the functional relevance of our genomic approach and to provide causal evidence for roles of the selected TFs in tumorigenesis, we examined how their inhibition affects the phenotype of *ras^V12^scrib^1^* tumors. While control larvae pupated on day 6-7 after egg laying (AEL), the majority of animals with EAD bearing *ras^V12^scrib^1^* clonal tumors arrested as third-instar giant larvae that ultimately died (Brumby and Richardson, 2003; Uhlirova and Bohmann, 2006). Only a few individuals formed pseudopuparia, starting on day 8 AEL (Fig. 3A). Consistent with previous reports (Uhlirova and Bohmann, 2006; Külshammer and Uhlirova, 2013), GFP-positive *ras^V12^scrib^1^* cells were highly invasive, penetrating the ventral nerve cord (VNC) of >80% of the developmentally arrested larvae (Fig. 3B).

Knockdown of *ets21c^LONG^* permitted nearly half of the animals to pupariate (Fig. 3A), but the *ras^V12^scrib^1^ets21c^LONG RNAi^* tumors remained highly invasive, infiltrating the VNC to an extent similar to *ras^V12^scrib^1^* tumors (Fig. 3B). Strikingly, inhibition of either Ftz-F1 or Fos (through a *kay^3^* mutant allele, *fos^RNAi^*, or overexpression of a JNK-phosphorylation site-deficient Fos^N-Ala^; Ciapponi et al., 2001) improved the pupation rate and suppressed tumor cell spreading into the VNC (Fig. 3A,B; supplementary material Fig. S3A,B; Uhlirova and Bohmann, 2006). This improvement did not result from a significant loss of tumor mass, as the tumor burden on day 6 AEL was similar between *ras^V12^scrib^1^*, *ras^V12^scrib^1^kay^3^* and *ras^V12^scrib^1^ets21c^LONG RNAi^* clones, and we observed only a slight reduction of GFP-positive tissue upon *ftz-f1* RNAi (Fig. 3C-F). Interestingly, this moderate tumor mass reduction coincided with a strong downregulation of an *expanded-lacZ* (*ex::lacZ*) reporter (Boedigheimer and Laughon, 1993; Hamaratoglu et al., 2005), indicating deregulation of the Hippo (Hpo) pathway and reduced Yorki (Yki) activity in the absence of Ftz-F1 (Fig. 3G). In contrast, *ex::lacZ* remained very active in EAD bearing *ras^V12^scrib^1^*, *ras^V12^scrib^1^bsk^DN^* or *ras^V12^scrib^1^kay^3^* clones (Fig. 3G; Külshammer and Uhlirova, 2013). Moreover, while loss of *fos* resulted in pupal lethality, 13% of the *ras^V12^scrib^1^ftz-f1^RNAi^* animals emerged as adults (Fig. 3H). Their compound eyes were larger than normal and rough on the surface (Fig. 3H), with fewer GFP-positive ommatidia compared with the controls (supplementary material Fig. S2D,E). Relative to *ras^V12^scrib^1^*, inhibition of Fos function markedly improved differentiation of photoreceptors and the overall morphology of the eye disc as revealed by staining against a pan-neuronal marker (ELAV) and Fasciclin III (FasIII), respectively (Fig. 3C,D; supplementary material Fig. S3C). Although elimination of *ets21c^LONG^* and *ftz-f1* slightly increased the number of GFP/ELAV double-positive cells, the normal ELAV pattern was still greatly disturbed (Fig. 3E′,F′). Unexpectedly, RNAi targeting the Fos-dimerizing partner Jun neither improved larval viability nor reduced tumor invasiveness or photoreceptor differentiation (Fig. 3A,B, data not shown), although *jun* RNAi reproduced previously reported phenotypes (Jindra et al., 2004; Sekyrova et al., 2010; supplementary material Fig. S3D) and depleted the Jun protein (supplementary material Fig. S3E,F).

**Fig. 4.**
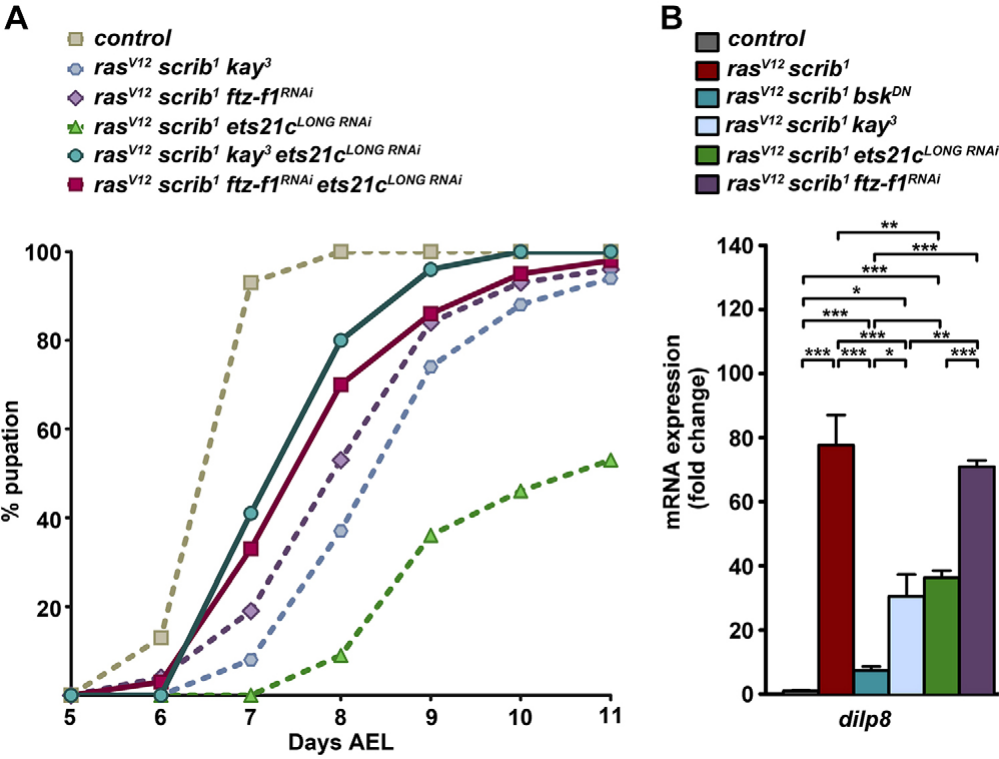
**Loss of *fos* or knockdown of *ets21c* partly suppresses *dilp8* expression, and simultaneous TF inhibition further improves pupation timing.** (A) Suppression of *ets21c^LONG^* and *fos* in *ras^V12^scrib^1^* tumors improved timing and progression of pupation by 1 day compared with the single knockdowns (*P*<0.0001; dashed lines repeated from Fig. 3). A mild improvement is also observed upon combined knockdown of *ets21c^LONG^* and *ftz-f1* (*P*<0.005)*.* Some *ras^V12^scrib^1^ftz-f1^RNAi^ets21c^LONG RNAi^* animals eclosed as adults, whereas *ras^V12^scrib^1^kay^3^ets21c^LONG RNAi^* individuals all died as pupae. The graph shows the cumulative percentage of pupae forming over time. (B) Elevated expression of *dilp8* mRNA in *ras^V12^scrib^1^* mosaic EAD was reduced upon JNK inhibition, loss of *fos* or *ets21c^LONG^* knockdown, but not in *ras^V12^scrib^1^ftz-f1^RNAi^* tumors. Data are mean values±s.e.m.; *n*=3-5; ***P<0.001; **P<0.005; *P<0.01.

To address whether candidate TFs cooperate during tumorigenesis, as suggested by our *in silico* analyses (Fig. 2A,B), we inhibited select TF pairs in the *ras^V12^scrib^1^* background. The simultaneous removal of *ets21c^LONG^* and *fos* rescued the timing and progression of pupation by 1 day compared with the single knockdowns (Fig. 4A). Nevertheless, these *ras^V12^scrib^1^kay^3^ets21c^LONG RNAi^* animals did not complete metamorphosis and all died as pupae. Interestingly, pupation of larvae bearing *ras^V12^scrib^1^ftz-f1^RNAi^ets21c^LONG RNAi^* clonal tumors was accelerated compared with *ras^V12^scrib^1^ftz-f1^RNAi^* and *ras^V12^scrib^1^ets21c^LONG RNAi^* larvae, and 13% of adults eclosed (Fig. 4A).

In summary, the data demonstrate that Ftz-F1 and Ets21c^LONG^ are both required for tumorigenesis because their depletion hindered development of fully malignant *ras^V12^scrib^1^* tumors, albeit to a different extent. We have shown that Ftz-F1 is essential for tumor invasiveness and tumor growth, the latter possibly through regulation of Hpo/Yki activity. We have further validated the requirement of Fos for *ras^V12^scrib^1^*-induced tumorigenesis whereas, surprisingly, the well-established Fos-dimerizing partner Jun appeared dispensable. We therefore suggest that Fos functions in *ras^V12^scrib^1^* tumors independently of Jun and describe a novel function for Fos in mediating differentiation defects of tumor clones. As simultaneous RNAi targeting of two TFs proved more efficient relative to single-gene interference, we conclude that cooperation among TFs of diverse families is a mechanism driving malignancy.

### Ets21c and Fos control *dilp8* expression

Recent studies have demonstrated that damaged or tumorous imaginal discs massively upregulate the *Drosophila* insulin-like peptide 8 (Dilp8), which delays pupariation by interfering with ecdysone production in the prothoracic gland (Colombani et al., 2012; Garelli et al., 2012). As larvae bearing *ras^V12^scrib^1^* tumors were able to pupariate upon loss of *fos* or knockdown of *ets21c* and even emerged as adults in the case of *ras^V12^scrib^1^ftz-f1^RNAi^* animals, we speculated that this improved viability might result from changes in *dilp8* expression. As expected, *dilp8* mRNA was highly elevated in *ras^V12^scrib^1^* mosaic EAD, and this increase was suppressed in EAD bearing *ras^V12^scrib^1^bsk^DN^* tumors (Fig. 4B, supplementary material Table S1). In *ras^V12^scrib^1^ets21c^LONG RNAi^* and *ras^V12^scrib^1^kay^3^* tumors, *dilp8* mRNA levels decreased significantly, although they still remained about 40-fold higher relative to control values. Remarkably, *dilp8* expression remained dramatically upregulated in *ras^V12^scrib^1^ftz-f1^RNAi^* mosaic EAD (Fig. 4B). Taken together, these data show that *dilp8* expression in malignant *ras^V12^scrib^1^* tumors requires JNK, and implicate Fos and Ets21c as JNK-regulated TFs contributing to high *dilp8* expression in these tumors.

### Ets21c acts as an oncogene in cooperation with Ras

Our data so far have demonstrated that Ets21c^LONG^, Ftz-F1 and Fos synergize downstream of JNK to promote tumor malignancy. Based on the phenotypes obtained with the single-TF knockdowns, Ftz-F1 and Fos seem to be more dominant players in *ras^V12^scrib^1^* tumors compared with Ets21c^LONG^. To test whether Ets21c^LONG^, Ftz-F1 or Fos may be sufficient to drive tumorigenesis, we overexpressed the individual TFs alone or in combination with Ras^V12^. Overexpression of Fos, Ets21c^LONG^, α*-*Ftz-F1 or β*-*Ftz-F1 alone did not noticeably alter the size, number or morphology of clones induced in the larval EAD (supplementary material Fig. S4A-E). Consistently, we did not observe upregulation of the well-established JNK target, MMP1 (Uhlirova and Bohmann, 2006), when the individual TFs were clonally expressed in the wing or eye/antennal imaginal disc (supplementary material Fig. S4F-I, data not shown).

**Fig. 5.**
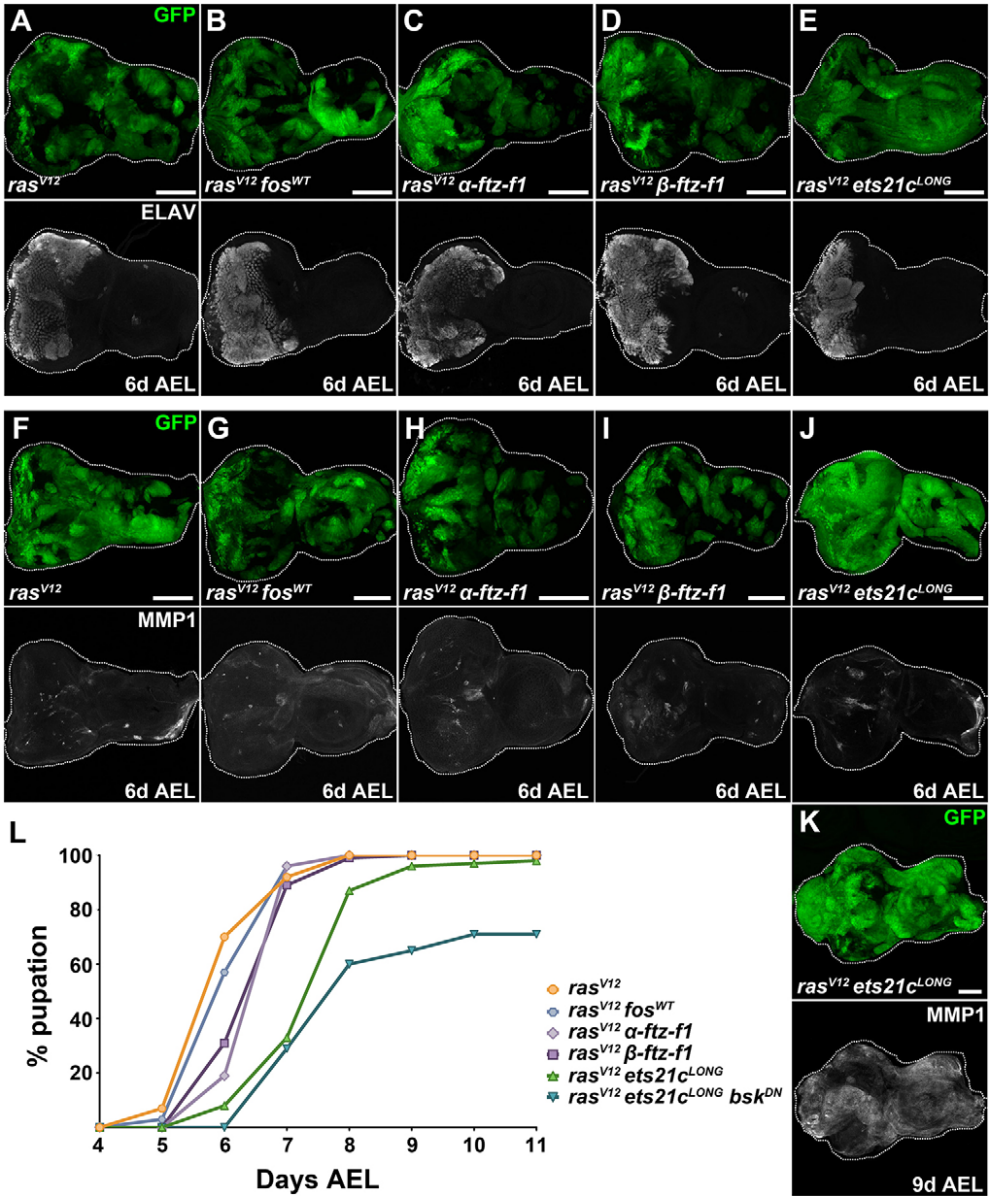
**Ets21c cooperates with Ras^V12^ to promote tumor growth, increase MMP1 expression and delay development.** (A-K) Co-expression of *ras^V12^* with *ets21c^LONG^* caused noticeable expansion of the GFP^+^ clonal area in EAD already on day 6 AEL (E,J). Nevertheless, photoreceptor differentiation marked by ELAV still occurred (E). Neither *fos^WT^* nor *α-* or *β-ftz-f1* overexpression was sufficient to enhance clonal tumor growth when combined with *ras^V12^* (B-D). Similar to *ras^V12^* mosaic EAD (F), clones co-expressing *ras^V12^* with *fos^WT^* (G), *α-ftz-f1* (H), *β-ftz-f1* (I) or *ets21c^LONG^* (J) showed only moderate enhancement of MMP1 levels on day 6 AEL. On day 9 AEL, *ras^V12^ets21c^LONG^* clones showed massive enrichment of MMP1 signal (K). Images show EAD as projections of multiple confocal sections. Scale bars: 100 µm (A-K). (L) *ras^V12^α-ftz-f1* and *ras^V12^β-ftz-f1* larvae pupated slightly later compared with *ras^V12^* alone or *ras^V12^fos^WT^* (*P<*0.0001). In contrast, pupation of *ras^V12^ets21c^LONG^* larvae was delayed by 2 days (*P<*0.0001). Inhibition of JNK (*ras^V12^ets21c^LONG^bsk^DN^*) further exacerbated the delay, arresting 29% of the tumor-bearing animals at the larval stage (*P<*0.0001). The graph shows the cumulative percentage of pupae forming over time.

Co-expression of either of the Ftz-F1 isoforms or Fos with Ras^V12^ resulted in phenotypes comparable to those described for *ras^V12^* alone; these mosaic EADs contained hyperplastic but non-invasive clonal tissue (Fig. 5A-D,F-I). The ELAV-positive domain was enlarged, and we detected only sporadic MMP1-labeled patches (Fig. 5A-D,F-I). The majority of larvae pupated at 6 days AEL and reached the P4 or P5 stage (Bainbridge and Bownes, 1981), at which they ultimately died (Fig. 5L). In contrast, larvae bearing *ras^V12^ets21c^LONG^* mosaic EAD were delayed, with the majority pupating at 7-9 days AEL compared with *ras^V12^* larvae (Fig. 5L).

**Fig. 6.**
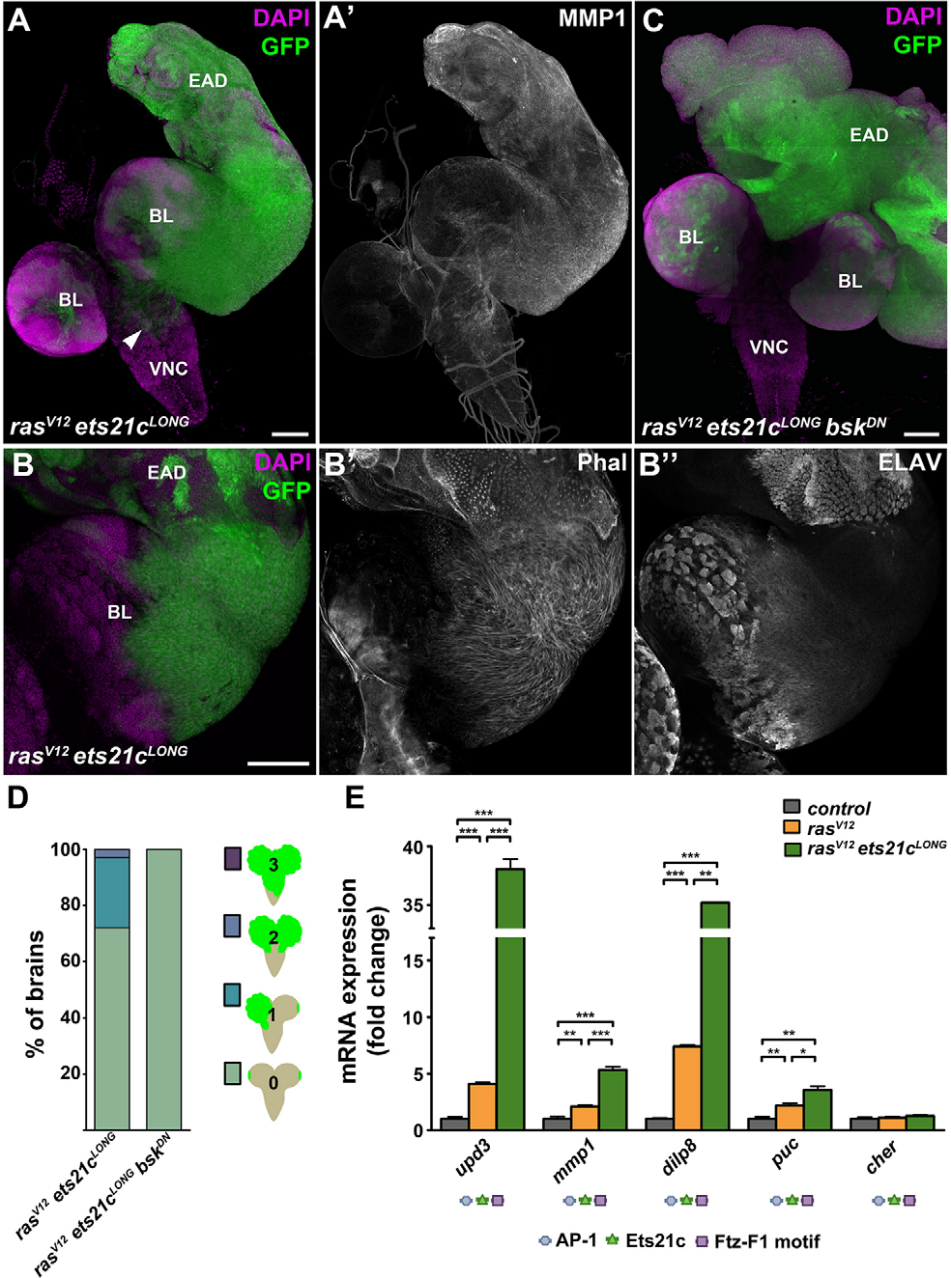
**Ets21c requires JNK activity to promote invasiveness but not growth of tumors.** (A-B) On day 9 AEL, *ras^V12^ets21c^LONG^* GFP-marked clones showed dramatic enrichment of MMP1 protein (A′) and filamentous actin, visualized with phalloidin (Phal; B′) in the cell cortex. Migrating cells were devoid of the differentiation marker ELAV (B″). *ras^V12^ets21c^LONG^* cells overgrew the entire EAD and spread over the brain lobes and VNC (arrowhead in A). (C) Blocking JNK (*ras^V12^ets21c^LONG^bsk^DN^*) suppressed tumor invasiveness but caused even greater overgrowth of GFP^+^ clonal tissue within the EAD. (D) Quantification of tumor invasiveness confirmed the requirement of JNK signaling for dissemination of *ras^V12^ets21c^LONG^* clonal cells. Four grades of invasiveness were scored based on spreading of clonal GFP-positive cells into larval brains dissected on day 8 AEL. Results are the percentage of brains in each category with *P*<0.0001. (E) *ras^V12^ets21c^LONG^* mosaic EAD showed marked increase in expression of the JNK targets *upd3*, *mmp1*, *dilp8* and *puc*, whereas *cher* expression was unaffected relative to control and *ras^V12^* mosaic EAD. Data are mean values±s.e.m.; *n*=3-5; ***P<0.001; **P<0.005; *P<0.01. Regulatory regions of all tested genes harbor AP-1, Ets21c and Ftz-F1 binding motifs. (A,C) show projections of multiple confocal sections, and (B) represents single sections. Scale bars: 100 µm (A-C). EAD, eye/antenna disc; BL, brain lobe; VNC, ventral nerve cord.

Dissection of *ras^V12^ets21c^LONG^* larvae on day 6 AEL revealed a noticeable enlargement of clonal tissue compared with EAD with *ras^V12^* clones alone (Fig. 5E,J). On days 8 and 9 AEL, the overall mass of *ras^V12^ets21c^LONG^* EAD increased dramatically and consisted almost exclusively of the clonal tissue that outcompeted the surrounding non-clonal cells (Figs 5K and 6A). Most strikingly, *ras^V12^ets21c^LONG^* cells were markedly enriched for MMP1 and filamentous actin (Figs 5K and 6A,B) and they efficiently invaded the brain lobes and the VNC (Fig. 6A,D). This enhanced invasiveness coincided with an inability to differentiate, as indicated by the absence of ELAV staining (Fig. 6B″).

In *ras^V12^ets21c^LONG^* animals, the development of aggressive tumors and the observed delay in pupariation were accompanied by transcriptional upregulation of some JNK target genes, namely the JNK phosphatase *puckered* (*puc*), the mitogenic cytokine *unpaired 3* (*upd3*), the pro-invasive *mmp1* and the pupation regulator *dilp8* (Fig. 6E). Expression of another established JNK target, the actin-crosslinker *cheerio* (*cher*), remained unchanged relative to control and *ras^V12^* backgrounds (Fig. 6E). The *cis*-regulatory regions of all of the above genes contain Ets21c binding sites (Fig. 6E, supplementary material Table S1). When ectopically expressed in the posterior compartment of the wing imaginal disc, Ets21c alone was sufficient to upregulate Dilp8-RFP and *puc-lacZ* reporters (supplementary material Fig. S5). Nevertheless, all of the examined genes contained AP-1 and Ftz-F1 motifs as well. It is therefore plausible that malignancy of *ras^V12^ets21c^LONG^* tumors arises from activation of JNK signaling through a positive feedforward loop, mediated by gain of Ets21c. To test the requirement of JNK signaling in tumorigenesis of *ras^V12^ets21c^LONG^* clones, we blocked JNK activity by overexpressing the dominant-negative form of Bsk. While the invasiveness of *ras^V12^ets21c^LONG^bsk^DN^* clones was clearly curbed (Fig. 6C,D), JNK inhibition did not suppress tumor growth (Fig. 6C) or improve the timing and rate of pupariation (Fig. 5L).

In conclusion, our results show that although Ftz-F1 and Fos are both required for invasiveness of *ras^V12^scrib^1^* tumors, these TFs were unable to promote malignant tumor overgrowth or MMP1 expression on their own or when combined with Ras^V12^. In contrast, Ets21c^LONG^ can, in cooperation with activated Ras, induce aggressive EAD clonal tumors that recapitulate the hallmarks of *ras^V12^scrib^1^* tumors. While invasiveness of *ras^V12^ets21c^LONG^* tumors requires JNK activity, the clonal overgrowth is JNK independent. Thus, in its tumor-promoting activity, gain of Ets21c^LONG^ can substitute for disrupted tissue polarity.

## DISCUSSION

Our genome-wide transcriptome profiling in the *Drosophila* epithelial tumor model has generated a comprehensive view of gene expression changes induced by defined oncogenic lesions that cause tumors of an increasing degree of malignancy. These data allowed us to discover how a network of collaborating transcription factors confers malignancy to *ras^V12^scrib^1^* tumors.

### Cooperating genetic lesions require JNK and control gene expression via a TF network

Our study revealed that the response of transformed *ras^V12^scrib^1^* epithelial cells is more complex in comparison to those with activated Ras^V12^ alone with respect to both the scope and the magnitude of expression of deregulated genes.

We have found that aberrant expression of more than half of the genes in *ras^V12^scrib^1^* tumors requires JNK activity, highlighting the significance of JNK signaling in malignancy. Importantly, the tumor-associated, JNK-dependent transcripts cluster with biological functions and processes that tightly match the phenotypes of previously described tumor stages (Pagliarini and Xu, 2003; Srivastava et al., 2007; Uhlirova and Bohmann, 2006; Leong et al*.*, 2009; Külshammer and Uhlirova, 2013). Furthermore, our *ras^V12^scrib^1^* transcriptome showed significant (*P<*0.0001) overlap (27% upregulated and 15% downregulated genes) with microarray data derived from mosaic EAD in which tumors were induced by overexpressing the BTB-zinc finger TF Abrupt (Ab) in *scrib^1^* mutant clones (Turkel et al., 2013) as well as with a transcriptome of *scrib^1^* mutant wing discs (35% upregulated and 18% downregulated genes; Bunker et al., 2015; supplementary material Fig. S6A,B). We propose that 429 misregulated transcripts (e.g. *cher*, *dilp8*, *ets21c*, *ftz-f1*, *mmp1*, *upd*), shared among all the three data sets irrespective of epithelial type (EAD versus wing disc) or cooperating lesion (Ras^V12^ or Ab), represent a ‘polarity response transcriptional signature’ that characterizes the response of epithelia to tumorigenic polarity loss (supplementary material Fig. S6C, Table S1). Our genome-wide profiling and comparative transcriptome analyses thus provide a foundation to identify novel candidates that drive and/or contribute to tumor development and malignancy while unraveling their connection to loss of polarity and JNK signaling.

In agreement with a notion of combinatorial control of gene expression by an interplay among multiple TFs (Miner et al., 1991; Elkon et al., 2003), we identified overrepresentation of *cis-*acting DNA elements for STAT, GATA, bHLH, ETS, BTB, bZIP factors and NRs in genes deregulated in *ras^V12^scrib^1^* mosaic EAD, implying that transcriptome anomalies result from a cross-talk among TFs of different families. Many of the aberrantly expressed genes contained binding motifs for AP-1, Ets21c and Ftz-F1, indicating that these three TFs may regulate a common set of targets and thus cooperatively promote tumorigenesis. This is consistent with the occurrence of composite AP-1-NRRE (nuclear receptor response elements), ETS-NRRE and ETS-AP-1 DNA elements in the regulatory regions of numerous human cancer-related genes, such as genes for cytokines, MMPs (e.g. stromelysin, collagenase) and MMP inhibitors (e.g. TIMP) (Miner et al., 1991; Kerppola et al., 1993; Li et al., 2000; Chinenov and Kerppola, 2001; Biddie et al., 2011).

Interestingly, *Drosophila ets21c* and *ftz-f1* gene loci themselves contain AP-1 motifs and qualify as polarity response transcriptional signature transcripts (supplementary material Table S1). Indeed, we have detected JNK- and Fos-dependent upregulation of *ets21c* and *ftz-f1* mRNAs in *ras^V12^scrib^1^* tumors (supplementary material Fig. S1). While JNK-mediated control of *ftz-f1* transcription has not been reported previously, upregulation of *ets21c* in our tumor model is consistent with JNK requirement for infection-induced expression of *ets21c* mRNA in *Drosophila* S2 cells and *in vivo* (Boutros et al., 2002; Radyuk et al., 2010; Chambers et al., 2012). Based on these data, we propose that Ftz-F1 and Ets21c are JNK-Fos-inducible TFs that together with AP-1 underlie combinatorial transcriptional regulation and orchestrate responses to cooperating oncogenes. Such an interplay between AP-1 and Ets21c is further supported by a recent discovery of physical interactions between *Drosophila* Ets21c and the AP-1 components Jun and Fos (Rhee et al., 2014). Whether regulatory interactions among AP-1, Ets21c and Ftz-F1 require their direct physical contact and/or the presence of composite DNA binding motifs of a particular arrangement to control the tumor-specific transcriptional program remains to be determined.

Importantly, some of the corresponding DNA elements, namely AP-1 and STAT binding sites, have recently been found to be enriched in regions of chromatin that become increasingly accessible in *ras^V12^scrib^1^* mosaic EAD relative to control (Davie et al., 2015). This demonstrates that comparative transcriptomics (present study) and open chromatin profiling using ATAC-seq and FAIRE-seq (Davie et al., 2015) are suitable complementary approaches for mining the key regulatory TFs responsible for controlling complex *in vivo* processes, such as tumorigenesis.

### Fos promotes tumor malignancy independently of Jun

The prototypical form of AP-1 is a dimer comprising Jun and Fos proteins. In mammals, the Jun proteins occur as homo- or heterodimers, whereas the Fos proteins must interact with Jun in order to bind the AP-1 sites (Kockel et al., 2001; Eferl and Wagner, 2003; Hess et al., 2004). In contrast to its mammalian orthologs, the *Drosophila* Fos protein has been shown to form a homodimer capable of binding to and activating transcription from an AP-1 element, at least *in vitro* (Perkins et al., 1990).

The role of individual AP-1 proteins in neoplastic transformation and their involvement in pathogenesis of human tumors remain somewhat elusive. While c-Jun, c-Fos and FosB efficiently transform mammalian cells *in vitro* (Jochum et al., 2001), only c-Fos overexpression causes osteosarcoma formation (Grigoriadis et al., 1993), whereas c-Jun is required for development of chemically induced skin and liver tumors in mice (Young et al., 1999; Eferl et al., 2003). In contrast, JunB acts as a context-dependent tumor suppressor (Passegue et al., 2001). Thus, cellular and genetic context as well as AP-1 dimer composition play essential roles in dictating the final outcome of AP-1 activity in tumors (Eferl and Wagner, 2003).

**Fig. 7.**
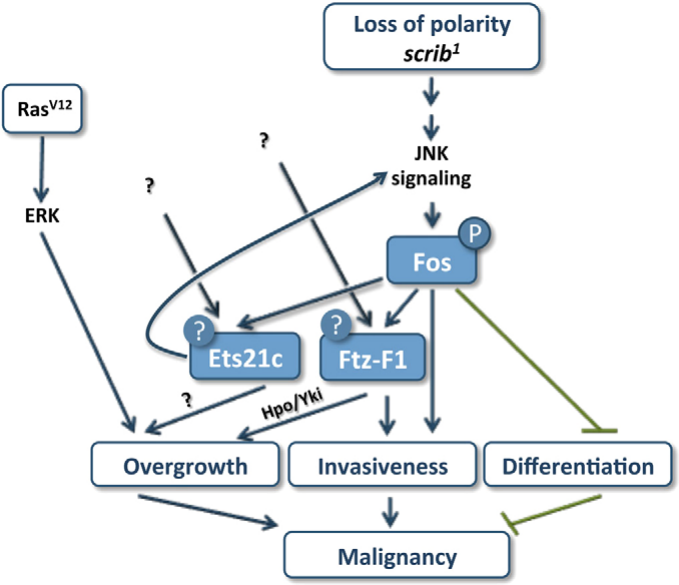
**A tripartite TF network drives tumor malignancy.** A model summarizing unique and common roles of Fos, Ets21c and Ftz-F1 in tumor malignancy that is provoked by oncogenic Ras signaling and the loss of the apico-basal polarity gene *scribble*. Fos and Ftz-F1 are both required for tumor invasiveness. While Fos prevents differentiation, Ftz-F1 contributes to tumor growth, possibly by deregulating Hpo/Yki signaling. Ets21c serves to fine-tune tumor gene expression. The oncogenic activity of Fos depends on its phosphorylation by JNK. Ets21c and Ftz-F1 are regulated transcriptionally, and unknown inputs additional to JNK are likely to control their activity. While Ets21c promotes tumor growth in a JNK-independent manner, Ets21c can uniquely substitute for loss of polarity and stimulate invasiveness through a feedforward loop, hijacking JNK activity.

Here, we show that, similar to blocking JNK with its dominant-negative form, Bsk (Igaki et al., 2006; Leong et al., 2009; Brumby et al., 2011; Külshammer and Uhlirova, 2013), removal of Fos inhibits *ets21c* and *ftz-f1* upregulation, suppresses invasiveness, improves epithelial organization and differentiation within *ras^V12^scrib^1^* tumors and allows larvae to pupate (Fig. 7 and Table 1). Strikingly, depletion of Jun had no such tumor-suppressing effects (Table 1). We therefore conclude that in the malignant *ras^V12^scrib^1^* tumors, Fos acts independently of Jun, either as a homodimer or in complex with another, yet unknown partner. A Jun-independent role for Fos is further supported by additional genetic evidence. Fos, but not Jun, is involved in patterning of the *Drosophila* endoderm (Szüts and Bienz, 2000) and is required for expression of specific targets, e.g. *misshapen* (*msn*) and *dopa decarboxylase* (*ddc*), during wound healing (Pearson et al., 2009; Lesch et al., 2010). Future studies should establish whether the JNK-responsive genes containing AP-1 motifs, identified in our study, are indeed regulated by Fos without its ‘canonical’ partner.

**Table 1.**
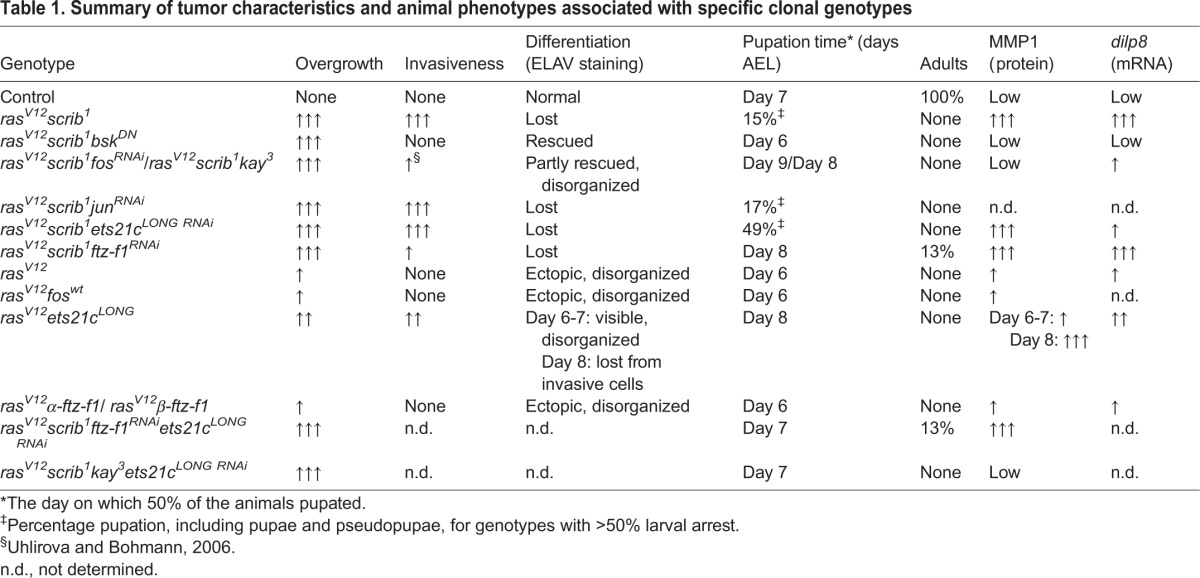
**Summary of tumor characteristics and animal phenotypes associated with specific clonal genotypes**

Our data identify Fos as a key mediator of JNK-induced MMP1 expression and differentiation defects in *ras^V12^scrib^1^* tumors. Only Fos inhibition caused clear suppression of MMP1 levels (supplementary material Fig. S7) and restoration of neurogenesis within clonal EAD tissue (Fig. 3C-F, supplementary material Fig. S3C), thus mimicking effects of JNK inhibition (Leong et al., 2009). Improved differentiation and reduced invasiveness are, however, not sufficient for survival of animals to adulthood, because interfering with Fos function in *ras^V12^scrib^1^* clones always resulted in pupal lethality.

### Ets21c and Ftz-F1 are novel mediators of JNK-driven malignancy with a unique and shared contribution to tumorigenesis

Our systems approach, followed by genetic experiments, identified Ets21c and Ftz-F1 as being essential for *ras^V12^scrib^1^*-driven tumorigenesis. We further show that mutual cooperation of both of these TFs with Fos is required to unleash the full malignancy of *ras^V12^scrib^1^* tumors (Fig. 7, Table 1).

TFs of the ETS-domain family are key regulators of development and homeostasis in all metazoans, whereas their aberrant activity has been linked with cancer (Sharrocks, 2001). *ets21c* encodes the single ortholog of human Friend leukemia insertion 1 (FLI1) and ETS-related gene (ERG) that are commonly overexpressed or translocated in various tumor types (Hsu and Schulz, 2000). While FLI1 is considered pivotal to development of Ewing's sarcoma (May et al., 1993), ERG has been linked to leukemia and prostate cancer (Yi et al., 1997; Petrovics et al., 2005). As for Ftz-F1 orthologs, the human liver receptor homolog-1 (LRH-1) has been associated with colonic, gastric, breast and pancreatic cancer (Annicotte et al., 2005; Schoonjans et al., 2005; Wang et al., 2008; Benod et al., 2011), whereas steroidogenic factor 1 (SF-1) has been implicated in prostate and testicular cancers (Straume et al., 2012; Lewis et al., 2014) and in adrenocortical carcinoma (Doghman et al., 2007). However, the molecular mechanisms underlying oncogenic activities of either the ERG/FLI1 or the SF-1/LRH-1 proteins are not well understood.

Here, we show that removal of Ftz-F1 markedly suppressed invasiveness of *ras^V12^scrib^1^* tumors, restoring the ability of tumor-bearing larvae to pupate. Additionally, and in contrast to Fos, Ftz-F1 inhibition also partly reduced tumor growth in the third-instar EAD and allowed emergence of adults with enlarged, rough eyes composed predominantly of non-clonal tissue (Fig. 7, Table 1). The reduced clonal growth coincided with downregulation of the well-established Yki target, *expanded*, implicating Ftz-F1 as a potential novel growth regulator acting on the Hpo/Yki pathway. We further speculate that reduced viability of *ras^V12^scrib^1^ftz-f1^RNAi^* clones and induction of non-autonomous compensatory proliferation by apoptotic cells during the pupal stage (Ryoo and Bergmann, 2012) could explain the enlargement of the adult eyes (Fig. 3H, supplementary material Fig. S2E). The precise mechanism underlying compromised growth and invasiveness of *ras^V12^scrib^1^ftz-f1^RNAi^* tumors and improved survival of the host remains to be identified.

In contrast, effects of Ets21c^LONG^ knockdown in *ras^V12^scrib^1^* tumors appeared moderate relative to the clear improvement conferred by either Fos or Ftz-F1 elimination. *ets21c^LONG RNAi^* neither reduced tumor mass nor suppressed invasiveness, and pupation was rescued only partly. However, unlike *ftz-f1^RNAi^*, *ets21c^LONG RNAi^* significantly reduced expression of *dilp8* mRNA. Based on abundance of Ets21c binding motifs in the regulatory regions of tumor-associated genes and the normalized expression of >20% of those genes upon removal of Ets21c, we further suggest that Ets21c acts in *ras^V12^scrib^1^* tumors to fine-tune the tumor gene-expression signature.

Dilp8 is known to be secreted by damaged, wounded or tumor-like tissues to delay the larval-to-pupal transition (Colombani et al., 2012; Garelli et al., 2012). We have corroborated the role of JNK in stimulating *dilp8* expression in *ras^V12^scrib^1^* tumor tissue, and further implicated Ets21c and Fos as novel regulators of *dilp8* downstream of JNK (Fig. 4B). However, our data also show that elevated *dilp8* transcription per se is not sufficient to delay metamorphosis. Unlike the permanent larvae bearing *ras^V12^scrib^1^* tumors, those with *ras^V12^scrib^1^ftz-f1^RNAi^* tumors pupated (Fig. 3A) despite the excessive *dilp8* mRNA (Fig. 4B). Likewise, pupation was not blocked by high *dilp8* levels in larvae bearing EAD clones overexpressing Abrupt (Turkel et al., 2013). As Dilp8 secretion appears critical for its function (Colombani et al., 2012), we propose that loss of Ftz-F1 might interfere with Dilp8 translation, post-translational processing or secretion.

Consistent with the individual TFs having unique as well as overlapping functions in specifying properties of *ras^V12^scrib^1^* tumors, knocking down pairwise combinations of the TFs had synergistic effects on tumor suppression compared with removal of single TF (Table 1). This evidence supports the view that malignancy is driven by a network of cooperating TFs, and elimination of several tumor hallmarks dictated by this network is key to animal survival. An interplay between AP-1, ETS-domain TFs and NRs is vital for development. For example, the ETS-factor Pointed has been shown to cooperate with Jun to promote R7 photoreceptor formation in the *Drosophila* adult eye (Treier et al., 1995). In mosquitoes, synergistic activity of another ETS-factor, E74B, with the ecdysone receptor (EcR/USP) promotes vitellogenesis (Sun et al., 2005). We thus propose that tumors become malignant by hijacking the developmental mechanism of combinatorial control of gene activity by distinct TFs.

### Gain-of-function experiments reveal an oncogenic potential of Ets21c

Despite the minor impact of *ets21c^LONG^* knockdown on suppressing *ras^V12^scrib^1^* tumors, Ets21c^LONG^ is the only one of the tested TFs that was capable of substituting for loss of *scrib* in inducing malignant clonal overgrowth when overexpressed with oncogenic Ras^V12^ in EAD. While invasiveness of such *ras^V12^ets21c^LONG^* tumors required JNK activity, JNK signaling appeared dispensable for tumor growth. Importantly, the overgrowth of *ras^V12^ets21c^LONG^* tumors was primarily independent of a prolonged larval stage, because we detected dramatic tumor mass expansion already on day 6 AEL. How cooperativity between Ets21c^LONG^ and Ras^V12^ ensures sufficient JNK activity and the nature of the downstream effectors driving tumor overgrowth remain to be determined. In contrast, co-expression of either Ftz-F1 or Fos with Ras^V12^ resulted in a non-invasive, Ras^V12^-like hyperplastic phenotype.

Why does Ets21c^LONG^ exert its oncogenic potential while Fos and Ftz-F1 do not? Simple overexpression of a TF may not be sufficient, because many TFs require activation by a post-translational modification (e.g. phosphorylation), interaction with a partner protein and/or binding of a specific ligand. Full activation of Fos in response to a range of stimuli is achieved through hyperphosphorylation by mitogen-activated protein kinases (MAPKs), including ERK and JNK (Ciapponi et al., 2001). Indeed, overexpression of a Fos^N-Ala^ mutated form that cannot be phosphorylated by JNK (Ciapponi et al., 2001) was sufficient to phenocopy *fos* deficiency, indicating that Fos must be phosphorylated by JNK in order to exert its oncogenic function. Consistent with our data, overexpression of Fos^N-Ala^ partly restored polarity of *lgl* mutant EAD cells (Zhu et al., 2010). We therefore conclude that the tumorigenic effect of Fos requires a certain level of JNK activation, which is lacking in EAD co-expressing Fos with Ras^V12^. Nevertheless, we cannot exclude the absence of an unknown Fos-interacting partner.

Interestingly, MAPK-mediated phosphorylation also greatly enhances the ability of SF-1 and ETS proteins to activate transcription (Wasylyk et al., 1998; Hammer et al., 1999). Two potential MAPK sites can be identified in the hinge region of Ftz-F1 (Pick et al., 2006), although their functional significance is unknown. Whether Ets21c or Ftz-F1 requires phosphorylation and how this would impact their activity in the tumor context remains to be determined. Our genetic experiments demonstrate that at least the overgrowth of *ras^V12^ets21c^LONG^* tumors does not require Ets21c phosphorylation by JNK.

In addition, previous crystallography studies revealed the presence of phosphoinositides in the ligand binding pocket of LHR-1 and SF-1 and showed their requirement for the NR transcriptional activity (Krylova et al., 2005; Blind et al., 2012). Although developmental functions of *Drosophila* Ftz-F1 seem to be ligand independent (Lu et al., 2013), it is still possible that Ftz-F1 activity in the tumor context is regulated by a specific ligand. We also cannot rule out an effect of Ftz-F1 SUMOylation (Talamillo et al., 2013).

### Concluding remarks

In summary, this work demonstrates that malignant transformation mediated by Ras^V12^ and *scrib* loss depends on MAPK signaling and at least three TFs of different families, Fos, Ftz-F1 and Ets21c. While their coordinated action ensures precise transcriptional control during development, their aberrant transcriptional (Ets21c, Ftz-F1) and/or post-translational (Fos, Ftz-F1, Ets21c) regulation downstream of the cooperating oncogenes contributes to a full transformation state. Our data implicate Fos as a primary nuclear effector of ectopic JNK activity downstream of disturbed polarity that controls *ets21c* and *ftz-f1* expression. Through combinatorial interactions on overlapping sets of target genes and acting on unique promoters, Fos, Ftz-F1 and Ets21c dictate aberrant behavior of *ras^V12^scrib^1^* tumors. Although originally described in *Drosophila*, detrimental effects of cooperation between loss of Scrib and oncogenic Ras has recently been demonstrated in mammalian tumor models of prostate and lung cancer (Pearson et al., 2011; Elsum et al., 2013). Our study and further functional characterization of complex TF interactions in the accessible *Drosophila* model are therefore apt to provide important insight into processes that govern cancer development and progression in mammals.

## MATERIALS AND METHODS

### Transgenic constructs

The coding sequence of *D. melanogaster ets21c-RA* (*ets21c^LONG^*) isoform was amplified from cDNA using Phusion polymerase (New England Biolabs; for primers see supplementary material Table S2) and cloned to *EcoRI* and *XhoI* of pENTR4 Dual Selection vector (Invitrogen, Carlsbad, CA, USA). The fragment was recombined into pTMW vector, enabling expression of the protein with N-terminal Myc tag (*Drosophila* Genomics Research Center), using the Gateway cloning system (Invitrogen, Carlsbad, CA, USA). Transgenic fly lines were obtained by random integration of the *UAS-Myc-ets21c^LONG^* transposon (Genetic Services).

### Fly strains and clonal analysis

The following fly strains were used: *UAS-ftz-f1^RNAi^* (#27659; Bloomington); *UAS-ets21c^RNAi^* (#106153; VDRC); *UAS-jun^RNAi^* (Jindra et al., 2004) *UAS-fos^35/19 RNAi^* (Hyun et al*.*, 2006); *UAS-fos^N-Ala^* (Ciapponi et al., 2001); *UAS-α-ftz-f1* and *UAS-β-ftz-f1* (Talamillo et al., 2013); *UAS-fos^WT^* (a gift from Dirk Bohmann, University of Rochester, Rochester, NY, USA); *kay^3^* (Külshammer and Uhlirova, 2013), *engrailed-GAL4*, *UAS-GFP* (Bloomington), *pnr-GAL4* (Calleja et al., 1996) and *Dilp8^(103492)^-CD8::RFP* (a gift from Alisson M. Gontijo, CEDOC, Oeiras, Portugal), *puc^E69^* (*puc-lacZ*; Martín-Blanco et al., 1998). To induce ‘flip-out’ clones (Struhl and Basler, 1993), progeny of *hsFLP; act>y^+^>GAL4*, *UAS-GFP/CyO* females crossed to males of desired genotype (supplementary material Table S3) were grown at 22°C. Recombination was induced by exposing larvae (3.5 days AEL) to heat shock at 37°C for 30 min, followed by incubation at 25°C before dissection at wandering third-instar larval stage. Generation of mosaics in the eye/antennal imaginal discs using the Mosaic analysis with a repressible cell marker method (MARCM; Lee and Luo, 2001) was carried out as described (Uhlirova et al., 2005) by crossing *ey-FLP1*; *act>y^+^>GAL4*, *UAS-GFP*; *FRT82B*, *Tub-GAL80* females to males of desired genotypes (supplementary material Table S3). MARCM fly crosses were carried out at 25°C on our standard media (Rynes et al., 2012).

### Quantification of tumor invasiveness and pupation rate

Tumor invasiveness was quantified as described previously (Külshammer and Uhlirova, 2013). For each genotype, a minimum of 65 EAD/brain complexes were analyzed. Statistical significance was determined using a χ^2^ test (Prism). Pupation rate was quantified by counting the number of pupal cases (prepupae and pupae) over time. Each graph represents the average of two to four independent experiments, including at least 34 individuals each. Statistical significance was determined using a log-rank test (Prism).

### Tissue staining

Tissues from third-instar larvae were processed as described previously (Külshammer and Uhlirova, 2013). The following primary and secondary antibodies were used at the indicated dilutions: mouse anti-MMP1 (mixture of 14A3D2, 3A6B4 and 3B8D12, 1:300), rat anti-ELAV (1:200; 7E8A10) and mouse anti-Fasciclin III (1:300, 7G10), all from Developmental Studies Hybridoma Bank (Iowa); and rabbit anti-Jun (1:500; present study). After washing, samples were incubated with a corresponding secondary antibody coupled to Cy3 or Cy5 (Jackson ImmunoResearch) for 2 h. Samples were counterstained with Alexa 546-phalloidin (Invitrogen) and DAPI to visualize actin filaments and nuclei, respectively. The *lacZ* activity was detected in imaginal discs using a standard X-Gal (5-bromo-4-chloro-3-indolyl-β-d-galactopyranoside) staining procedure described previously (Külshammer and Uhlirova, 2013).

### Image acquisition and processing

Confocal stacks were acquired at room temperature with an Olympus FV1000 confocal microscope equipped with 20× UPlan S-Apo (NA 0.85), 40× UPlanFL (NA 1.30) and 60× UPlanApo (NA 1.35) objectives. Maximal projections were generated using Fluoview 2.1c software (Olympus) and ImageJ (Abramoff et al., 2004). Final image processing, including panel assembly, brightness and contrast adjustment, was done in Photoshop CS5.1 (Adobe Systems, Inc.). *Z*-stacks of adult eyes were taken using a motorized Leica M165 FC fluorescent stereomicroscope equipped with a DFC490 CCD camera. Images were processed using the Multifocus module of LAS 3.8.0 software (Leica). White outlines of the EAD shown in figures were drawn based on staining with DAPI.

### Quantitative reverse transcription-PCR (qRT-PCR)

Total RNA was isolated from mosaic EAD at 6 days AEL with Isol-RNA Lysis Reagent (5 Prime), and 2 µg of DNase-treated RNA was transcribed using Superscript III reverse transcriptase with oligo (dT) primers (Life Technologies). Quantitative RT-PCR was performed with SYBR green mix (Bio-Rad, Hercules, CA, USA) using the CFX96 (Bio-Rad) real-time PCR system. All qRT-PCR primers (supplementary material Table S2) were designed to anneal at 62°C. Data were normalized to *rp49* transcript levels, and fold changes in gene expression were calculated using the relative standard curve method (Larionov et al., 2005). At least four biological replicates were analyzed per experiment. Statistical significance was determined using Student's unpaired two-tailed *t*-test with unequal variance.

### Messenger RNA expression profiling by next-generation sequencing

RNA was isolated and DNase treated as stated above. Sequencing libraries generated according to the Illumina protocol for total RNA library preparation were pair-end sequenced on an Illumina HiSeq 2000 instrument at 100 bp read length. Image analysis and base calling were done with the Illumina RTA software at run time. Data were processed using a high-throughput next-generation sequencing analysis pipeline. Basic read quality check was performed with FastQC (v0.10.1), and read statistics were acquired with SAMtools v0.1.19 (Li et al., 2009). Reads were mapped to the *Drosophila* reference assembly (version BDGP R5/dm3, April 2006) using Tophat v2.0.10 (Trapnell et al., 2009), and gene quantification was carried out using a combination of Cufflinks v2.1.1 (Trapnell et al., 2010) and the DESeq2 package v1.4.5 (Anders and Huber, 2010), with genomic annotation from the Ensembl database, version 75. The results were uploaded into an in-house MySQL database and joined with BiomaRt v2.20.0 (Durinck et al., 2005) annotations from Ensembl, version 75. Lists of differentially expressed genes were defined by a final database export using 5 and 0.01 as cut-offs for DESeq2-based FCs and *P*-values, respectively. To identify genes differentially expressed in the respective conditions, the average of at least two biological replicates was calculated. Supplementary material Table S1 shows all transcripts whose expression differed ≥1.5-fold in *ras^V12^scrib^1^* compared with the control (*FRT82B*). The expression of ‘Rescued’ genes changed ≥1.5-fold with respect to *ras^V12^scrib^1^* in the direction of the control. ‘Opposite’ genes changed ≥1.5-fold compared with the control but in the opposite direction to *ras^V12^scrib^1^.* The FlyBase Gene Ontology (GO) terms were used for functional annotation, and DAVID allowed gene ontology clustering (http://david.abcc.ncifcrf.gov/home.jsp; Huang et al., 2009). All raw next generation sequencing data are available from the GEO database (accession number GSE65261). To determine the overlap between different gene-expression data sets, all genes that changed ≥1.5-fold compared with the control were considered. Fisher's exact test was adopted to calculate the significance of the intersection between the data sets.

### *In silico* analysis of TF binding motifs

Cytoscape 3.1.1 with iRegulon plugin v1.2 was used to search for overrepresented TF binding sites among genes regulated in different tumor genotypes (Shannon et al., 2003; Janky et al., 2014). The 5 kb upstream region, the 5′ untranslated region and the first intron of each regulated gene were considered under default iRegulon settings with the receiver operating characteristic (ROC) threshold for calculation of area under the cumulative recovery curve (AUC) adjusted to 3%. The selected position weight matrices for AP-1 (FBgn000129 kay_Jra_SANGER_5), Ets21c (FBgn0005660 Ets21c_SANGER_5) and Ftz-F1 (FBgn0001078 ftz-f1_FlyReg) were used with FIMO to search for motif occurrence in the first intron and 5 kb upstream sequence of all genes differentially regulated in the *ras^V12^scrib^1^* transcriptome (Bailey et al., 2009; Grant et al., 2011). The corresponding genome regions (version BDGP R5/dm3, April 2006) are available from the GEO database (accession number GSE65261). The results were visualized in Cytoscape 3.1.1.
